# Intra-arterial delivery of triolein emulsion increases vascular permeability in skeletal muscles of rabbits

**DOI:** 10.1186/1751-0147-51-30

**Published:** 2009-07-16

**Authors:** Hak Jin Kim, Yong Woo Kim, In Sook Lee, Jong Woon Song, Yeon Joo Jeong, Seon Hee Choi, Kyung Un Choi, Kuen Tak Suh, Byung Mann Cho

**Affiliations:** 1 The Department of Radiology, Pusan National University College of Medicine, Medical Research Institute, Pusan National University, Pusan, South Korea; 2 The Department of Radiology, Yangsan Pusan National University Hospital, Yangsan, South Korea; 3 The Department of Radiology, Paik Hospital, Inje University, Pusan, South Korea; 4 The Department of Paracytology, Pusan National University College of Medicine, Pusan, South Korea; 5 The Department of Pathology, Pusan National University College of Medicine, Pusan, South Korea; 6 The Department of Orthopedic Surgery, Pusan National University College of Medicine, Pusan, South Korea; 7 The Department of Preventive Medicine, Pusan National University College of Medicine, Pusan, South Korea

## Abstract

**Background:**

To test the hypothesis that triolein emulsion will increase vascular permeability of skeletal muscle.

**Methods:**

Triolein emulsion was infused into the superficial femoral artery in rabbits (triolein group, n = 12). As a control, saline was infused (saline group, n = 18). Pre- and post-contrast T1-weighted MR images were obtained two hours after infusion. The MR images were qualitatively and quantitatively evaluated by assessing the contrast enhancement of the ipsilateral muscles. Histologic examination was performed in all rabbits.

**Results:**

The ipsilateral muscles of the rabbits in the triolein group showed contrast enhancement, as opposed to in the ipsilateral muscles of the rabbits in the saline group. The contrast enhancement of the lesions was statistically significant (p < 0.001). Histologic findings showed that most examination areas of the triolein and saline groups had a normal appearance.

**Conclusion:**

Rabbit thigh muscle revealed significantly increased vascular permeability with triolein emulsion; this was clearly demonstrated on the postcontrast MR images.

## Background

The vascular endothelium serves as a barrier with protective properties. This barrier can, however, be an obstacle to drug delivery. As emulsified triolein or fatty acids change the vascular permeability of the brain [1,2], the testis [3], and the orbit [4], a fat emulsion model may be useful in studies regarding methods of drug delivery. However, as there have only been a few studies [1-4] using a fat emulsion model, further experiments using this technique are still needed on various types of vessels and organs. On the other hand, fatty acids have been reported to be more toxic than triolein in the lung [5-8] and brain [9]. In a study of increased vascular permeability induced by an infusion of triolein emulsion into the brain [1], MR images reverted to normal signal intensity within 4 days. We therefore hypothesized that an emulsified triolein could be useful in a vascular permeability study of the skeletal muscle since significant pathologic changes did not occur in the brain.

There are two main types of capillaries: 1) the fenestrated type, as exists in the liver and 2) the continuous type, as exists in the brain, orbit, skin, and muscle. In contrast to the capillaries of the brain or the orbit, the muscle capillaries are surrounded with relatively loose connective tissue [10]. The vascular endothelium is a simple squamous epithelium that has acquired a remarkably high permeability to water and water soluble solutes, including macromolecules, through a characteristic process of differentiation of its cells. This differentiation includes numerous plasmalemmal vesicles. There is evidence that these vesicles function as mass-carriers of fluid and solutes across the endothelium and as generators of trans-endothelial channels by concomitant fusion with both domains (luminal and tissue) of the plasmalemma. The endothelial fenestrae of visceral capillaries are initially transendothelial channels subsequently collapsed to a minimal length. The intercellular junctions of the capillary endothelium are not permeable to tracers of diameter >18 – 20 A.

Two main components are recognized in the analysis of capillary permeability: a basic component comparable to that of other simple epithelia and involving transport across the plasmalemma and probably along the intercellular junctions (for molecules of diameter > 10 A); and a differentiated component, which involves plasmalemmal vesicles and their derivatives, i.e., transendothelial channels and fenestrae [11]. Experimental triolein or oleic acid embolism has been studied mainly in the brain and the testis. Triolein-induced vascular change in skeletal muscle has not yet been reported. Such a study would, however, be helpful in investigating the pathophysiologic mechanism of the effect of triolein, not only on capillaries with a barrier such as in the brain, the retina or the testis, but also on capillaries without a barrier such as in the skeletal muscle. The capillaries of muscle are permeable to plasma proteins [12-14]. Thus they are different from the capillaries of the brain. The type of capillaries of muscle is called as a non-BBB-type [15]. The purpose of this study was to investigate the changes in vascular permeability of skeletal muscle caused by triolein emulsion, by means of contrast-enhanced MR imaging.

## Methods

### Preparation of the Rabbits

The Animal Research Committee of the Medical Research Institution approved all of our experiments and surgical procedures. A total of 30 New Zealand white rabbits (Samtako, Osan, Korea), weighing 3.0 – 3.5 kg each, were used in the present study. All rabbits were anesthetized with intramuscularly administered ketamine HCl (2.5 mg/kg; Korea United Pharm, Seoul, South Korea) and xylazine (0.125 mg/kg; Bayer Korea, Seoul, South Korea) into the shoulder muscles, and were ventilated with room air. The body temperature was measured using a rectal probe (MGA-III 219, Shibaura Electronics, Tokyo, Japan) and was maintained at 35.5–36.5°C with a heating pad.

Following anesthetization of each rabbit, the left common carotid artery was isolated and its distal portion was ligated with 4.0 silk. An 18-gauge catheter (Insyte; Becton Dickson Vascular Access, Sandy, UT, USA) was inserted into the artery, and a 3.0F micro-catheter (MicroFerret-18 Infusion Catheter; William Cook Europe, Bjaeverskov, Denmark) with a micro-guidewire was passed through the catheter into the lumen of the artery. Under fluoroscopic guidance, the micro-catheter tip was positioned in the left superficial femoral artery. The thigh muscles were chosen as a model for investigating the effect of emulsified fat on a non-penetrating artery because of their easy accessibility with fluoroscopic guidance and the high quality of the MR imaging due to the bulkiness of these muscles.

### Injection of Triolein Emulsion

Triolein emulsion was injected following the technique of Kim et al. [1]. A 1-mL syringe containing 0.2 mL of neutral triglyceride triolein (Sigma-Aldrich, St. Louis, MO, USA) and a 25-mL syringe containing 20 mL of saline (1% triolein solution) were connected to a three-way stopcock. In a previous study of emulsified triolein on the blood-brain barrier [1], the dose of triolein was 0.1 mL; however, as the volume of thigh muscle is larger than that of the brain, the amount of triolein was doubled. The triolein emulsion was made by mixing via the stopcock with vigorous to-and-fro movement of the syringes for 2 minutes. Triolein globules ranged in size from 1 to30 μm, with most < 2 or 3 times larger than red blood cells [1]. In 12 rabbits (triolein group), the emulsified fat was infused manually into the superficial femoral artery at a rate of 4 mL/minute for 5 minutes. As a control (saline group, n = 18), 20 mL normal saline rather than triolein emulsion was infused into the superficial femoral artery using the same technique at a rate of 4 mL/minute for 5 minutes.

### MR imaging

Pre- and post-contrast T1-weighted MR imaging (1.5T, Sonata, Siemens, Erlangen, Germany) of the thigh was performed 2 hours after the triolein emulsion injection in triolein group or normal saline injection in saline group. The imaging time was based on the fact that there is maximum contrast enhancement approximately 2 hours after triolein embolization [9,16]. The rabbits were placed in a supine position on a homemade wooden table, and a flexible coil was placed above both thighs. Images were acquired in the axial plane. For T1-weighted imaging, the following parameters were used: TR/TE of 985/21 ms; section thickness of 4 mm with a 0.1-mm gap; FOV of 90 mm; two excitations; and an acquisition matrix of 256 × 179. For contrast studies, 0.2 mmol/kg gadopentate dimeglumine (Magnevist, Schering, Germany) was injected via the auricular veins. One minute after contrast injection, postcontrast MR imaging was performed.

### MR Image Analysis

For qualitative evaluation of vascular permeability changes, pre- and postcontrast T1-weighted images were analyzed for the presence and pattern of abnormal signal intensities or enhancement in the thigh muscles in both groups. For quantitative evaluation of vascular permeability changes, the signal intensity (SI) was measured on the pre- and post-contrast T1-weighted images using a round region of interest (ROI; range, 7 – 8 cm2) in the adductus magnus muscle to the mid-femoral shaft on five continuous images in the emulsion treated thigh, and the mean value was obtained in both groups. Contrast enhancement ratios (CERs = SI post-contrast/SI pre-contrast – 1) were measured using raw data of the SIs of the pre- and post-contrast T1-weighted images of both groups. The significance of the differences in the CERs between the triolein group and the saline group was estimated using the Mann-Whitney U test, and a p-value < 0.05 was considered significant.

### Histologic Examination

Immediately after MR imaging, the rabbits were sacrificed by using sodium thiopental. For light microscopic examination with hematoxylin-eosin stain, the ipsilateral muscle was obtained in the adductor magnus of the mid-level of the thigh in both groups, according to the contrast enhancing area on MR images. Edema, hemorrhage, and necrosis were evaluated. For electron microscopic examination, three areas of the harvested muscle were selected. These areas were cut into 1 mm cubes for the preparation of electron microscopy blocks. The samples were prefixed with 2.5% glutaraldehyde in phosphate-buffered saline at pH 7.2 for 2 h at 1–40C and washed in 0.1 M phosphate-buffered saline. Next, the samples were fixed in 1% OsO4 solution for 2 h and washed in the 0.1 M phosphate-buffered saline. After washing, samples were dehydrated with alcohol, mordanted en bloc overnight with poly/Bed 812 resin (Polysciences, Warrington PA, USA), and stored for 12 h at 37°C, followed by 48 h at 45°C. Resin-embedded blocks were cut into sections, 1 μm in thickness, stained with toluidine blue, and then the areas of interest were selected under a light microscope. Ultrathin sections were prepared using an ultramicrotome (Leica, Vien, Austria) with a diamond knife and applied to nickel 150 mesh grids. Samples were stained with uranyl acetate and lead citrate, and examined with a transmission electron microscope (JEM 1200 EX-II; JEOL, Tokyo, Japan). The presence of intravascular or extravascular triolein emulsion, the integrity of the space, and interstitial edema were evaluated.

## Results

### Qualitative Analyses

**Figure 1:**
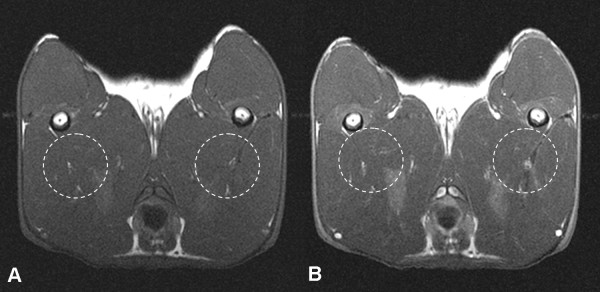
**Pre-contrast (A) and post-contrast (B) T1-weighted axial images (TR/TE, 985/21) of a rabbit obtained 2 hours after normal saline injection into the left superficial femoral artery (saline group) revealed minimal contrast enhancement of the thigh muscles around the ipsilateral femur**. The contrast enhancement is the same as that observed in the contralateral femur. White circular dots present regions of interest where signal intensity was measured in the ipsilateral and contralateral adductor magnus muscles.

**Figure 2:**
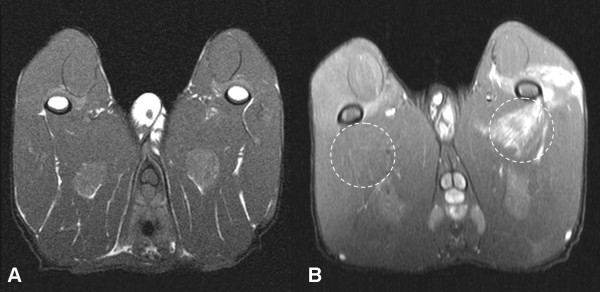
**Pre-contrast (A) and post-contrast (B) T1-weighted axial image (TR/TE, 985/21) of a rabbit obtained 2 hours after triolein emulsion into the left superficial femoral artery (triolein group) reveals focal enhancement of the thigh muscles posterior (adductor magnus) and lateral (vastus lateralis and vastus intermedius) to the ipsilateral femoral bone**. White circular dots present regions of interest where signal intensity was measured in the ipsilateral and contralateral adductor magnus muscles.

In the control group (saline group), minimal or no contrast enhancement was visualized on the post-contrast T1-weighted MR images (Fig. 1). In all rabbits in the triolein group, the muscles of the ipsilateral thigh showed abnormal focal or diffuse contrast enhancement (Fig. 2).

### Quantitative Assessments

SIs on the pre-contrast T1-weighted images of the triolein group were similar to those of the saline group [mean SIs (standard deviation) of the triolein and saline groups: 504.1 (56.48) and 582.2 (519.1), respectively; number of measurements = 12]. However, the thigh muscles of the triolein group showed remarkably increased SIs on the post-contrast T1-weighted images compared with those of the saline group [mean SIs (standard deviation) of the triolein and saline groups: 1793.0 (229.94) and 711.8 (704.01), respectively; number of measurements = 18; Table 1). The difference in CERs between groups 1 and 2 was significant (p < 0.001, two-tailed p-value).

**Table 1:** Mean Signal Intensities (Sis) and Mean Contrast Enhance Ratios (CERs) on Pre- and Post-contrast T1-weighted MR Images of the Triolein and Saline Groups

	Pre-contrast	Post-contrast	CER*
Triolein Group (n = 12)	504 (56.5)	1793 (229.9)	2.61 (0.73)
Saline Group (n = 18)	582 (519.1)	712 (704.0)	0.22 (0.37)

*: p < 0.001

### Histologic Findings

**Figure 3:**
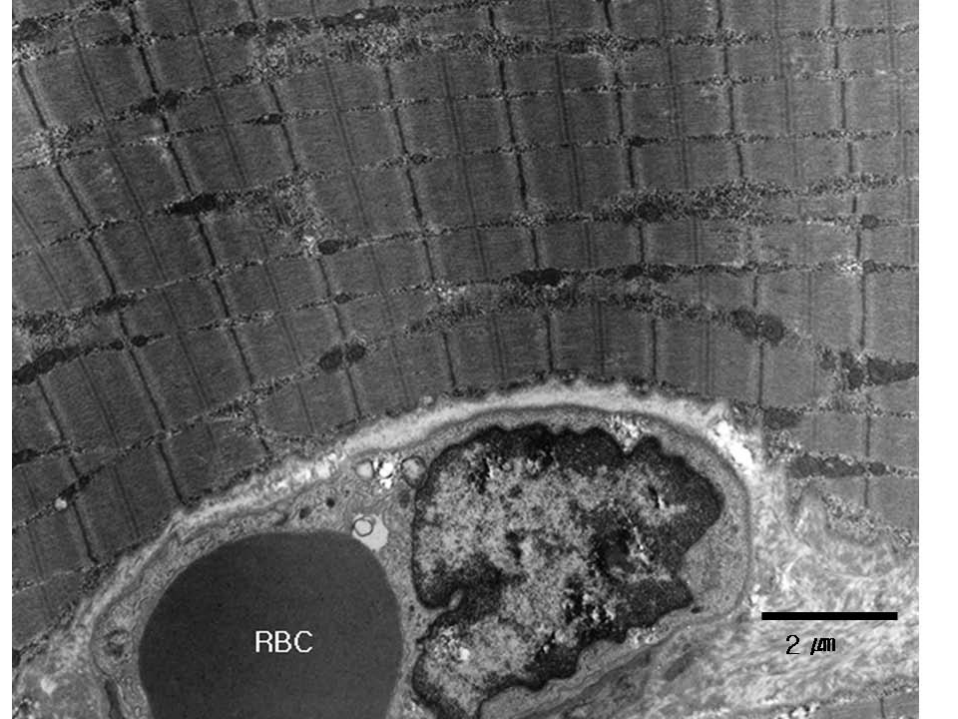
**Electron micrograph of skeletal muscle obtained from the adductor magnus of the ipsilateral thigh in a rabbit in the saline group (original magnification × 4000)**. An endothelial cell and an intraluminal red blood cell (RBC) is seen at the bottom of the image. Longitudinal section of the muscle shows orderly arranged A bands and Z lines. No interstitial edema or disruption of the endothelium is noted. bar: 2 μm

**Figure 4:**
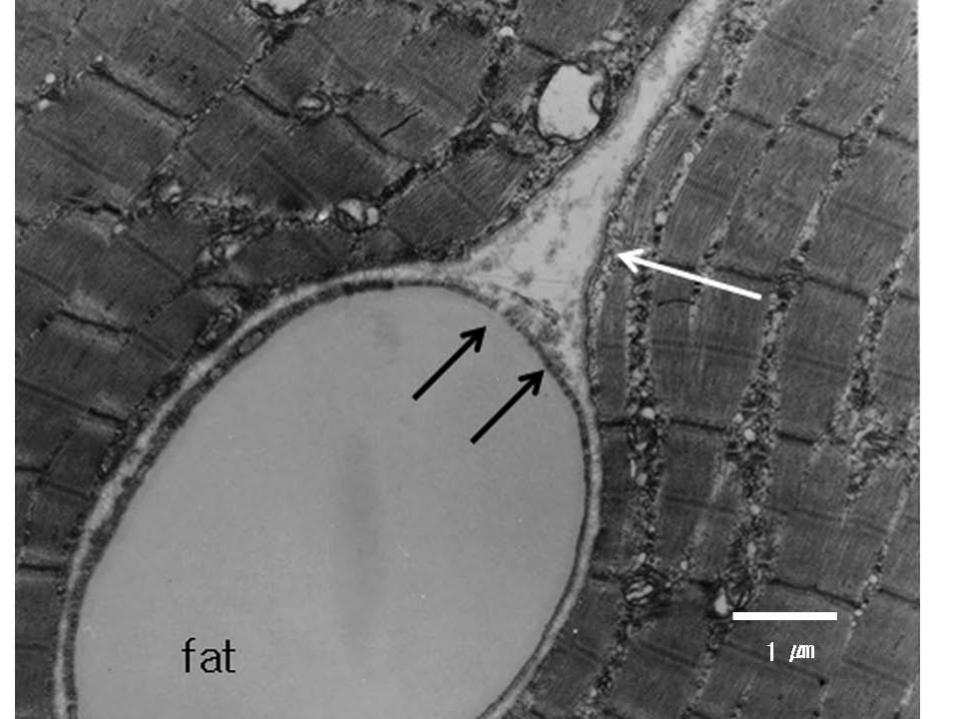
**Electron micrograph of skeletal muscle obtained from the adductor magnus of the ipsilateral thigh in a rabbit in the triolein group (original magnification × 5000)**. The capillary is enlarged due to an impacted fat globule (Fat). Longitudinal section of the muscle reveals no evidence of disruption of the A bands or Z lines. Minimal disruption of the upper portion of the endothelium (black arrows) with minimal interstitial edema (white arrow) is seen. bar: 1 μm

On light microscopy, most of the examined areas in the triolein group had a normal appearance, similar to that of the saline group. Necrosis or hemorrhage was not evident in either group. On electron microscopy, most portions of the ipsilateral thigh muscle in the triolein group showed no significant changes compared to the saline group (Fig. 3). Intravascular fat globules were visualized sporadically in 11 rabbits in the triolein group. The capillaries containing the fat globules showed a dilated lumen. A defect of the endothelial wall of capillaries and minimal interstitial edema were noted in three rabbits in the triolein group (Fig. 4). However, the defect was focal and infrequent in each field. The nuclei of the endothelial cells in the triolein group did not differ from those in the saline group.

## Discussion

In the present study, the muscles of the ipsilateral thigh of the rabbits in the triolein group were significantly enhanced on post-contrast T1-weighted images compared with the rabbits in the saline group. This result demonstrates that emulsified triolein increases vascular permeability. In a study of vascular permeability, the signal intensity of a brain lesion was 92% higher than the contralateral hemisphere on contrast-enhanced T1-weighted images obtained two hours after a bolus injection of 0.1 mL triolein [17]. In the present study, the SI of the ipsilateral thigh muscle was 260% higher in the triolein group than the control group on post-contrast T1-weighted images. It is difficult to compare the quantitative results of Kim et al. [17] with our results because of the different organs studied and the different amounts (0.1 mL in the former study and 0.2 mL in the current study) and status (bolus triolein and oleic acid in the former study and triolein emulsion in the current study) of the triolein used. However, whether the vascular permeability changes induced by triolein are due to a direct impact on the integrity of the lipoproteinaceous layers of the endothelial walls has not been proven.

Fat (such as subcutaneous fat or lipomas) shows high signal intensity on T1-weighted image. In the present study, however, too small amount of triolein (0.2 ml) was used in an emulsified state to reveal any hyperintensity on T1-weighted image [1,3].

The capillaries of skeletal muscles have both similar and different characteristics compared to the brain. The walls of the blood capillaries of the skeletal muscles of the hind legs of rabbits consist of three consecutive layers or tunics, i.e., the endothelium (inner layer), the basement membrane with its associated pericytes (middle layer), and the adventitia (outer layer) [18]. Of these three layers, the endothelium regulates the passage of proteins and colloidal particles across the capillary wall [19-22]. The pericytes, related to growing capillary sprouts by synthesis of the extracellular matrix components [23], are numerous in the cerebral cortex although, by contrast, they are rare in the muscle [10,24]. The muscles and central nervous system have continuous endothelium containing numerous small pinocytotic vesicles (diameter, 50 – 70 nm) along both their luminal and basal surfaces [25]. Cerebral capillaries are always completely and closely invested by neuropils (networks of naked nerve fibers and processes of astrocytes), in marked contrast to the very loose connective tissues in the vicinity of muscle capillaries [10].

All membranes, including those of the endothelial cells, are composed primarily of lipid and protein together with a small amount of carbohydrate. Membrane lipids, mostly phospholipids, have a hydrophilic phosphate (polar) end and a hydrophobic, non-polar end (fatty acid tail). Membrane proteins are globular and float like icebergs in a sea of lipids [26]. The plasma membrane presents a lipid barrier to the passage of some substances and is partly determined by their lipid solubility. Larger molecules can enter a cell by the process of pinocytosis, i.e., a local invagination of the plasma membrane enclosing fluid and then pinching off to form a membrane-bound vesicle in the cell. The ability to transfer substances through the walls of capillaries is referred to as permeability. Permeability varies regionally and under changing conditions. In continuous capillaries, it is generally accepted that the vesicles or caveolae participate in carrying metabolites, and perhaps fluid across the capillary walls. The basal lamina does not present a major barrier to the passage of most substances or to the formed elements of blood [26].

Chan et al. [27] proposed hypothetical mechanisms for the development of brain edema caused by fatty acids. Initially, this development induced by various pathologic insults begins with activation of phospholipases A2 and/or C. These enzymes hydrolyze membrane phospholipids, thereby forming arachidonic acid and other lipid compounds. Arachidonic acid is readily converted into prostaglandin, thromboxanes, and oxygen-free radicals by cyclooxygenase. Free radicals and arachidonic acid induce structural disturbances of the cellular membranes of various target cells. The membrane perturbation of neurons and glia induced by arachidonic acid causes both reduction in the uptake of the neurotransmitters, GABA and glutamate, as well as a reduction in Na+, K+-ATPase activity. The membrane perturbation may also activate the vesicular transport of macromolecules across brain endothelial cells (pinocytosis). On the other hand, opening of the blood-brain barrier may also result from damage to endothelial cells. Thus, the increased permeability of solutes and water from blood to brain leads to the development of vasogenic edema. The mechanism of triolein on blood vessels is still unclear. Triolein is indicated to be the precursor of free fatty acids. Therefore, the effect of triolein and free fatty acids on blood vessels would be similar, in part, because triolein is changed to a free fatty acid by the action of lipases [28]. However, precisely when triolein is converted to a free fatty acid in tissue is unknown. In addition, the effect of fatty acids has been shown to be more toxic in the brain and lung compared to the effect of triolein [5-9,17,29]. Therefore, triolein might have a different mechanism in the blood vessels compared with free fatty acids. Of all elements, gadolinium has the strongest influence on T1 relaxation times of hydrogen protons [30]. Chelating gadolinium to DTPA reduces, but far from eliminates, gadolinium's strong influence on proton T1 and T2 relaxation. The very high hydrophilicity, the charge, and the rather large molecular weight of Gd-DTPA (about 550) probably accounts for its exclusion by biologic barriers, such as cell membranes [31]. Gadolinium-enhanced MR imaging provides a minimally-invasive means of mapping barrier breakdown, while also allowing other disease-related phenomena to be studied. This technique, which exploits the T1-shortening effect produced by the leakage of a contrast agent through a damaged barrier into the extravascular space has been used extensively to provide qualitative delineation of regional BBB abnormalities. While it also enables serial, qualitative assessments of barrier function to be made on a regional basis, the possibility of making quantitative measurements of vessel wall permeability provides a potentially powerful tool in the study of a barrier opening in a number of clinical pathologies [32].

The present light-microscopic study showed no significant changes in the triolein group compared with the saline group. Ultrastructurally, however, intravascular fat globules occurred sporadically in the triolein group. The capillaries containing the fat globules had a dilated lumen, and in three rabbits, the endothelium of capillaries containing fat globules showed small focal defects of the wall in some areas. The impaction of the fat globules was thought to be due to their larger size compared with the size of the capillary lumen. In the present study, the triolein emulsion was made by manual to-and-fro movements through a narrow passage of two syringes. Thus, the size of the fat globules was not homogenous, as some might be larger than the capillary lumen. The size of the fat globules that was most harmful to the endothelial wall was not proven in the present study. In further studies it will be important to obtain a more homogenous size of the globules in the triolein emulsion.

The results of the present study could not explain the pathophysiological mechanism of the effect of emulsified triolein on the endothelial wall, and they could not tell whether increased contrast media permeability is also relevant to other molecules, such as drugs. Disruption of a protein binding mechanism, lipoproteinaceous layer disorientation, or both could be related to the vascular permeability changes. Molecular-based studies, which were beyond the scope of the present study, could help to understand the underlying mechanism. Increased contrast media permeability with emulsified triolein may be applicable to the increased drug permeability and should be studied further by performing radioisotope studies.

## Conclusion

The vascular permeability of the skeletal muscles of the thigh increased with infusion of a triolein emulsion into the superficial femoral artery in the present study. This increased vascular permeability was revealed as contrast enhancement on the post-contrast MR images. These results can be helpful in understanding the mechanism of triolein emulsion on the vessel wall and the related pathology occurring in skeletal muscles. The triolein emulsion model can also be used in research regarding drug delivery in order to evaluate the adjuvant effect for chemotherapy.

## Competing interests

The authors declare that they have no competing interests.

## Acknowledgments

This study was supported by Medical Research Institute Grant (2006-65), Pusan National University.

## Authors' contributions

HK has made substantial contributions to conception and design, YK carried out acquisition of data, IL analysed data, JS interpreted data, YJ involved in drafting the manuscript, SC acquired data, KC interpreted pathologic images, KS conceived of the study, BC performed the statistical analysis. All authors read and approved the final manuscript.
